# Elucidating the role of ubiquitination and deubiquitination in osteoarthritis progression

**DOI:** 10.3389/fimmu.2023.1217466

**Published:** 2023-06-09

**Authors:** Chenxiao Zheng, Jiayi Chen, Yurui Wu, Xiaochao Wang, Yongan Lin, Lilu Shu, Wenjun Liu, Peter Wang

**Affiliations:** ^1^Department of Orthopaedics and Traumatology, Zhongshan Hospital of Traditional Chinese Medicine Affiliated to Guangzhou University of Traditional Chinese Medicine, Zhongshan, Guangdong, China; ^2^Department of Orthopaedics, The Second Clinical Medical College of Guangzhou University of Chinese Medicine, Guangzhou, Guangdong, China; ^3^South China University of Technology, Guangzhou, Guangdong, China; ^4^Department of Medicine, Zhejiang Zhongwei Medical Research Center, Hangzhou, Zhejiang, China

**Keywords:** ubiquitination, deubiquitination, osteoarthritis, E3 ligase, DUBs

## Abstract

Osteoarthritis is non-inflammatory degenerative joint arthritis, which exacerbates disability in elder persons. The molecular mechanisms of osteoarthritis are elusive. Ubiquitination, one type of post-translational modifications, has been demonstrated to accelerate or ameliorate the development and progression of osteoarthritis *via* targeting specific proteins for ubiquitination and determining protein stability and localization. Ubiquitination process can be reversed by a class of deubiquitinases *via* deubiquitination. In this review, we summarize the current knowledge regarding the multifaceted role of E3 ubiquitin ligases in the pathogenesis of osteoarthritis. We also describe the molecular insight of deubiquitinases into osteoarthritis processes. Moreover, we highlight the multiple compounds that target E3 ubiquitin ligases or deubiquitinases to influence osteoarthritis progression. We discuss the challenge and future perspectives *via* modulation of E3 ubiquitin ligases and deubiquitinases expression for enhancement of the therapeutic efficacy in osteoarthritis patients. We conclude that modulating ubiquitination and deubiquitination could alleviate the osteoarthritis pathogenesis to achieve the better treatment outcomes in osteoarthritis patients.

## Introduction

Osteoarthritis (OA) is one common type of non-inflammatory degenerative arthritis, which is a joint disease and influences older persons for walk, leading to disability in elder persons (1, 2). Pain is the primary symptom in osteoarthritis patients. The features of osteoarthritis are the destructions of cartilage extracellular matrix (ECM), loss of mobility, joint dysfunction and synovial inflammation (3, 4). Aging and injure are two common reasons for the development of osteoarthritis. The exact mechanisms of osteoarthritis are not fully understood, but it is thought to involve a complex interplay of genetic, environmental, and biomechanical factors (5–9). The treatments often include pain control and reduction of inflammation (10). It is of great importance to discover the mechanisms of osteoarthritis and develop the novel therapeutic strategies for osteoarthritis (11, 12).

In general, protein is synthesized from the genetic code in DNA and translated into a polypeptide chain. Protein could be modified *via* the chemical changes, which is named as post-translational modification (PTM) (13, 14). PTMs are found to modulate protein functions *via* an effect on various protein behavior, such as activity, localization, stability and interactions with other proteins (15). Many common PTM types include methylation (addition of a methyl group), acetylation (addition of an acetyl group), ubiquitination (attachment of a small ubiquitin protein), sumoylation (attachment of the small protein SUMO), glycosylation (addition of sugar molecules) and phosphorylation (addition of a phosphate group), which affect localization, stability, interaction, folding, and gene expression (16–19). PTMs are critical in governing some biological processes, such as cell signaling pathways, cell metabolism, differentiation, metastasis, cell cycle and proliferation (20).

**Figure 1:**
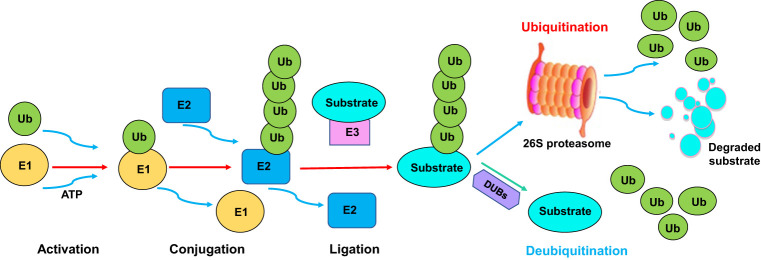
The ubiquitination and deubiquitination processes. The ubiquitination process is performed by E1 ubiquitin-activating enzyme, E2 ubiquitin-conjugating enzyme, and E3 ubiquitin ligase. E1 enzymes activate ubiquitin and transfer it to E2 enzyme. Then, E2 enzymes interact with E3 enzymes, leading to the transfer of ubiquitin from the E2 enzymes to the specific target proteins. The ubiquitinated proteins can be recognized and degraded by the proteasome. Ubiquitination can be reversed by deubiquitination, which is a process of cleaving ubiquitin from target proteins. Deubiquitination is carried out by a class of deubiquitinases (DUBs).

Ubiquitination is one type of PTMs, which involves the covalent attachment of ubiquitin to a target substrate. Ubiquitination affects substrate protein functions *via* regulation of localization and stability (21). The ubiquitination process is performed by E1 ubiquitin-activating enzyme, E2 ubiquitin-conjugating enzyme, and E3 ubiquitin ligase. E1 enzymes activate ubiquitin and transfer it to E2 enzyme. Then, E2 enzymes interact with E3 enzymes, leading to the transfer of ubiquitin from the E2 enzymes to the specific target proteins (22, 23). The ubiquitinated proteins can be recognized and degraded by the proteasome machinery (24, 25). Hence, the E3 ligases determine the specificity of the ubiquitination reaction due to recognizing and interacting with specific substrates (**Figure 1**). Several types of E3 ligases are RING (really interesting new gene) ligases, HECT (homologous to E6-AP carboxyl terminus) ligases, RBR (RING-between-RING) ligases, U-box ligases and PHD-finger ligases (26, 27). Ubiquitination can be reversed by deubiquitination, which is a process of cleaving ubiquitin from target proteins (28) (**Figure 1**). Deubiquitination is carried out by a class of deubiquitinases (DUBs), including ovarian tumor proteases (OTUs), ubiquitin-specific proteases (USPs), ubiquitin C-terminal hydrolases (UCHs), and Josephin domain-containing proteins (29). Among these DUBs, USPs are the largest family to cleave ubiquitin from substrates. Dysregulation of USPs have been implicated in various diseases, such as neurodegeneration, inflammation and cancer (30–32). It is clear that the balance between ubiquitination and deubiquitination is pivotal for maintaining proper protein levels and their functions (33).

In recent years, ubiquitination and deubiquitination have been found to play a potential role in the various pathologies, including osteoarthritis (34). In this review, we will describe the role of ubiquitination in the development and progression of osteoarthritis. Moreover, we discuss the function of deubiquitination in regulating osteoarthritis development. Furthermore, we describe the compounds that target ubiquitination and deubiquitination to influence the osteoarthritis progression. Lastly, we provide the challenge and future perspectives for targeting ubiquitination and deubiquitination to treat with osteoarthritis patients.

## The role of ubiquitination in osteoarthritis

### NEDD4 E3 ubiquitin ligases in osteoarthritis

NEDD4 E3 ubiquitin ligases are a family of enzymes that share a C2 domain at N-terminal and HECT domain at C-terminal, which regulate protein degradation, membrane trafficking and signaling transduction (35). This family includes several members, such as NEDD4, NEDD4-2, WWP1, WWP2, ITCH, Smurf1, Smurf2, NEDL1 and NEDL2. NEDD4 family has been involved in regulation of various diseases, including cancer, inflammation, and osteoarthritis (36–38). In the following paragraphs, we will discuss the mechanisms of NEDD4 E3 ubiquitin ligases in osteoarthritis initiation and progression (**Table 1**).

**Table 1:** E3 ubiquitin ligases regulate osteoarthritis development and progression.

Name	Targets	Functions	Ref
WWP1	N/A	Involves in osteoarthritis development	(39)
WWP2	Runx2	Modulates osteoarthritis	(40)
Smurf1	N/A	Regulates osteoarthritis	(41)
Smurf2	SIRT1	Regulates apoptosis, proliferation, inflammation in osteoarthritis chondrocytes	(42)
ITCH	JAG1	Regulates chondrocyte damage and cartilage damage	(43)
FBXW7	MKK7	Regulates chondrocyte degeneration and osteoarthritis	(44)
FBXO3	IL-18, IL-1β, pyroptosis-related proteins	Regulates knee osteoarthritis	(45)
FBXO6	MMP14	Controls post-injury osteoarthritis development	(46)
FBXO21	ERK	Regulates osteoarthritis-associated cartilage degeneration	(47)
HECTD1	Rubicon	Controls stress-induced chondrocyte death and osteoarthritis	(48)
Cbl-b	TrkA	Regulates osteoarthritis pain	(49)
UFL1	NO, PGE2, iNOS, COX-2, MMP-3, MMP-13, ADAMTS-4, ADAMTS-5	Involves in osteoarthritis development	(50)
HRD1	OS9	Regulates osteoarthritis development	(51)

### WWP1

WWP1 (WW domain-containing protein 1), an E3 ubiquitin ligase, plays an essential role in regulating protein degradation, cellular signal transduction, and gene expression. WWP1 targets proteins and make them for degradation by the proteasome (52). Recent studies have implied that WWP1 may involve in the development and progression of osteoarthritis. One bioinformatics analysis using cartilage samples showed that several genes are potential biomarker of osteoarthritis, such as WWP1, MDM2, OAS2, MSH2, UBE2E3, BCL2, RB1, TYMS and EGFR (39). In addition, miR-5692, miR-548e-5p and miR-3613-3p were associated with the abovementioned genes. It is required to determine whether WWP1 could be a useful biomarker of osteoarthritis (39).

### WWP2

WWP2 (WW domain-containing protein 2) belongs to the NEDD4 family of E3 ubiquitin ligases. WWP2 contains several domains, such as a C2 domain, a HECT domain, and multiple WW domains. These domains are responsible for transferring the ubiquitin to target proteins (53). WWP2 controls the protein stability and biological functions *via* providing ubiquitin molecules to targets, resulting in proteasomal degradation or modulation of protein activity or changing protein subcellular localization (54). Due to that WWP2 is involved in a wide range of biological functions, dysregulation of WWP2 leads to various disease states, such as neurodegeneration, cancer, inflammation and viral infections (55, 56). WWP2 was involved in chondrogenesis and osteoarthritis by regulating cartilage-specific transcription factors (57). WWP2 overexpression led to an inhibition of COL2A1, STC2, ACAN, GDF10 and GJA1, and an upregulation of EPAS1 expression using 3D chondrocyte pellet culture system. In addition, miR-140 overexpression caused an increased WWP2 and WDR1 in chondrocytes (58).

Yang et al. reported that miR-140 co-expressed with WWP2-C isoform, which can be accelerated by Sox9. Silencing of miR-140 reduced chondrogenic proliferation *via* targeting Sp1 (59). NFAT3 (nuclear factor of activated T-cells protein 3) and TGF-β/SMAD3 (mothers against decapentaplegic homolog 3) controlled miR-140 expression in osteoarthritis (60). Depletion of NFAT5 inhibited the expression of miR-140 and WWP2. NFAT3 and SMAD3 can bind to miR-140, but depletion of NFAT3 reduced miR-140 expression without altering WWP2 (60). Mice without WWP2 and mice with inactivation type of WWP2 E3 ligase (WWP2-C838A) had spontaneous and induced osteoarthritis. WWP2 regulated the expression of Runx2 *via* poly-ubiquitination and degradation and reduced Adamts5, contributing to cartilage homeostasis. WWP2 mRNA injection attenuated the severity of osteoarthritis in mouse joints (40). One methylation quantitative trait loci (mQTLs) analysis showed that WWP2 was linked to osteoarthritis genetic risk (61). In a word, WWP2 is involved in modulation of osteoarthritis.

### SMURF1 and SMURF2

Smad ubiquitin regulatory factor 1 (Smurf1) and Smurf2 belong to the family of NEDD4 ubiquitin ligase. Both Smurf1 and Smurf2 are involved in regulation of protein levels in the ubiquitin-dependent manner, which control the cell cycle progression, cell growth and differentiation (62). Although Smurf1 and Smurf2 share many functional similarities, they have distinct roles in governing various cellular processes to maintain cellular homeostasis. Dysregulation of Smurf1 and Smurf2 has been linked to cancer, inflammation and developmental disorders (63–65). One group used chondrogenic progenitor cells (CPCs) and meniscus progenitor cells (MPCs) and found that both Smurf1 and Smurf2 existed in articular cartilage and meniscus. An increased expression of Smurf1 alleviated the levels of TGFBR1, RUNX2 and SOX9, while upregulation of Smurf2 inhibited the levels of RUNX2 and GFFBR1, but not SOX9 levels. Depletion of Smurf2 increased the levels of SOX9 and RUNX2 in CPCs, but not in MPCs. Inhibition of Smurf1 did not affect the levels of RUNX2, SOX9 and TGFBR1 (41). Smurf1 protein was upregulated more in mice with Smurf2 depletion than wildtype mice upon TFG-β3 stimulation (66). Smurf2 has been observed to regulate osteoarthritis *via* targeting β-catenin in cartilage (67). Smurf2 was strongly expressed in human osteoarthritis compared with nonarthritic cartilage. Mice with Smurf2 overexpression displayed less articular cartilage area, osteophytes, clefting, subchondral sclerosis, and fibrillation. These mice exhibited the high expression of MMP-13 and type X collagen in articular cartilage. Moreover, Smurf2-transgenic mice had a decreased TGF-β pathway and an increased degradation of pSmad3 (68). Mice with Smurf2 deficiency exhibited higher expression of SOX, Col2 and Acan compared with wildtype mice (66). Chondrocytes with Smad3 deficiency accelerated differentiation and activated BMP signaling pathway, but failed to increase the expression of Smurf2, Sno, TGFRII in chondrocytes (69).

Increased expression of Smurf2 in cells of the chondrogenic lineage blocked differentiation of chondrocytes and enhanced maturation as well as promoted osteoblast differentiation. This phenotype could be due to upregulation of beta-catenin in the chondrocytes after Smurf overexpression, indicating that Smurf2 might be responsible for the development of osteoarthritis (70). One study showed that Smurf2 mediated degeneration of articular cartilage *via* promoting degradation of GSK-3beta and induction of beta-catenin in chondrocytes (71). LncRNA TM1-3P targeted miR-144-3p and ONECUT2 (one cut homeobox 2), governed cell apoptosis, inflammation and proliferation in osteoarthritis (72). LncRNA-CRNDE depletion elevated the ubiquitination of SIRT1 induced by SMURF2, resulting in reduction of SIRT1 stability. Overexpression of lncRNA-CRNDE stimulated the interaction of collagen 2 promoter and SOX9 (42). Circ_0116061 regulated the expression of miR-200b-3p and SMURF2, leading to modulation of cell apoptosis, proliferation, and inflammation in osteoarthritis chondrocytes (73). Lead, an environmental toxin, impaired TGF-β signaling pathway *via* upregulation of Smurf2 and downregulation of pSmad2 and pSmad3, leading to osteoarthritis in articular chondrocytes (74).

### ITCH

ITCH (also known as AIP4), a member of NEDD4 family of E3 ubiquitin ligases. It is pivotal to regulate signaling pathways and immune system *via* degradation of different proteins (75–77). One group reported that mice with ITCH deficiency in macrophages exhibited more severe joint damage compared with wildtype mice after post-traumatic osteoarthritis surgery. Mice with ITCH deficiency displayed promotion of the inflammatory macrophage infiltration in the synovium (78). ITCH protein levels were decreased during post-traumatic osteoarthritis. Macrophages with ITCH depletion were stimulated with IL-1β and exhibited pro-inflammatory phenotype. Hence, ITCH attenuated the progression of post-traumatic osteoarthritis *via* suppression of macrophage polarization (78). Another group identified that ITCH alleviated LPS-mediated chondrocyte injury through regulating JAG1 ubiquitination in osteoarthritis (43). ITCH expression was decreased in osteoarthritis samples compared with normal cartilaginous samples, whereas JAG1 expression was elevated in osteoarthritis specimens. Upregulation of ITCH or depletion of JAG1 induced proliferation, attenuated inflammation and ECM degradation and blocked apoptosis in LPS-mediated chondrocytes (43). Specifically, ITCH interacted with JAG1 *via* the WW-PPXY motif and caused JAG1 degradation *via* K48-linked ubiquitination, leading to inactivation of Notch1 pathway. Upregulation of JAG1 restrained the ITCH-mediated protection of chondrocyte damage that was induced by LPS. In conclusion, LPS-mediated chondrocyte injury and osteoarthritis-mediated articular cartilage damage were mitigated by ITCH in part *via* inhibition of Notch1 activation by enhancement of JAG1 ubiquitination and degradation (43).

### F-box proteins in osteoarthritis

The F-box protein is one of the subunits of the Skp1-Cullin1-F-box (SCF) complex, which acts as the substrate recognition component (79). The F-box protein contained a conserved F-box motif, which binds to a scaffold protein Skp1 to provide the link for the F-box protein to the rest of the SCF complex. Cullin1 interacts with Skp1 and F-box protein to form a core structure of the SCF complex. It is important to note that the variable region of F-box proteins determines its specificity for various substrates, leading to the ubiquitination of specific target proteins and degradation by the proteasome (80). F-box proteins regulate cell growth, apoptosis, differentiation, autophagy, cell cycle and metastasis. Abnormal expression of F-box proteins could participate in the development of cancer, inflammation and neurodegenerative disorders (81–83). For example, FBXO45 targeted GGNBP2 for ubiquitination and degradation and enhanced progression of esophageal squamous cell carcinoma (84). In the next paragraphs, we will mention the function of numerous F-box proteins in governing osteoarthritis initiation and progression (**Table 1**).

### FBXW7

FBXW7 (F-box and WD repeat domain-containing 7) has been well studied in regulation of various cellular processes. Numerous targets of FBXW7 have been identified, including c-Myc, c-Jun, Notch, cyclin E and Mcl-1 (85). Trough targeting these proteins, FBXW7 controls cell proliferation, survival, cell cycle, differentiation and apoptosis. Mutations in FBXW7 were reported in various human malignancies. In tumorigenesis, FBXW7 has been revealed to be a tumor suppressive protein (86, 87). In recent years, FBXW7 was characterized to take part in osteoarthritis development. Mechanical overloading led to acceleration of senescence in chondrocyte and in mouse articular cartilage, which accompanied with downregulation of FBXW7 in chondrocytes and mouse cartilage (44). In line with this finding, FBXW7 was decreased in cartilage tissues in ageing mice, osteoarthritis mice, osteoarthritis patients. Depletion of FBXW7 in chondrocytes enhanced cartilage catabolism and caused chondrocyte senescence *via* promotion of p16, p21 and Colx and reduction of Col2a1 and ACAN, contributing to the exacerbation of osteoarthritis (44). On the contrary, overexpression of FBXW7 by intra-articular injection of adenovirus restrained osteoarthritis in mice. Further experiments indicated that mechanical overloading reduced the transcription of FBXW7 and alleviated FBXW7-induced degradation of MKK7, leading to activation of JNK signaling pathway (44).

Circular RNA VMA21 (circVMA21) expression was impeded by IL-1β in chondrocytes and C28/I2 cells. Ectopic expression of circVMA21 elevated the expression of Bcl-2 and reduced Bax and C-caspase-3 in C28/I2 cells after IL-1β stimulation, resulting in viability promotion and apoptosis attenuation (88). In addition, upregulation of circVMA21 reduced the MMP1 and MMP13 expressions, elevated COL2A1 expression and attenuated the production of NO, PGE2, TNF-α and IL-1β. Mechanistically, circVMA21 sponged miR-495-3p and consequently upregulated the expression of FBXW7, a target of miR-495-3p in chondrocytes (88). Zhu et al. reported that FBXW7 participated in IL-1β-involved chondrocytes degeneration *via* modulation of HIF-1α and VEGF pathways (89). In particular, lower expression of FBXW7 and higher expression of VEGF and HIF-1α were observed in osteoarthritis cartilage and IL-1β-mediated degenerated chondrocytes. Inhibition of HIF-1α led to reduction of VEGF levels, leading to upregulation of aggrecan, SOX9 and collagen II, and downregulation of collagen I and Runx-2 expression. Therefore, FBXW7 impeded HIF-1α/VEGF pathway and displayed a protective function in IL-1β-mediated chondrocyte degeneration *via* altering the expression of collagen I, collagen II, SOX9, aggrecan and Runx-2 (89).

### FBXO3

FBXO3 has been found to stimulate cytokine secretion *via* controlling the degradation of Fbxl2 and subsequent upregulation of TNFR-associated factor (TRAF) proteins (90). FBXO3 was also reported to potentiated vascular inflammation and atherosclerosis (91). One study used rat fibroblast-like synoviocytes (FLSs) to construct knee osteoarthritis cell model. This cell model exhibited elevation of IL-18, IL-1β, apoptosis rate, and increased expression of pyroptosis-associated proteins. The abovementioned indicators were downregulated by up-modulation of miR-219a-5p, while downregulation of miR-219a-5p exhibited the opposed results (45). FBXO3 expression was inhibited by miR-219a-5p, and depletion of FBXO3 repressed the expression of IL-18, IL-1β and pyroptosis-related proteins. FBXO3 counteracted the effects of miR-215a-5p-inhibited IL-18, IL-1β and pyroptosis-related proteins. Additionally, miR-219a-5p alleviated knee joint injury and elevated step size of rat with knee osteoarthritis partly *via* inhibition of FBXO3 and inactivation of NLRP3 (45).

### FBXO6

Wang et al. explored the effects of FBXO6 on the osteoarthritis pathogenesis. The human osteoarthritis samples and several mouse osteoarthritis models were used to detect the expression of FBXO6. This study showed that FBXO6 was downregulated in the cartilage from aged mouse samples, spontaneous osteoarthritis samples, ACLT (anterior cruciate ligament transaction)-induced osteoarthritis samples (46). Osteoarthritis process was accelerated in mice with cartilage conditional knockout or global knockout of FBXO6. FBXO6 reduced the MMP14 protein levels *via* ubiquitination and degradation, contributing to inactivation of MMP13 proteolytic ability. Notably, TGF-β-SMAD2/3 axis can upregulate the expression of FBXO6. Overall, upregulation of FBXO6 attenuated post-injury osteoarthritis development, suggesting that induction of FBXO6 in cartilage could be a useful strategy for treating osteoarthritis (46).

### FBXO21

Lin et al. evaluated the effects of FBXO21 in osteoarthritis degeneration and its underlying molecular mechanism. They reported that osteoarthritis patient cartilages had higher expression of FBXO21. Ageing rats and monosodium iodoacetate-driven osteoarthritis rats exhibited an increased expression of FBXO21 in cartilages (47). FBXO21 expression was elevated in chondrocytes after treatment with lipopolysaccharide, IL-1β and TNF-α. Additionally, downregulation of FBXO21 alleviated osteoarthritis-associated cartilage degeneration, which accompanied with promotion of autophagy and anabolism, reduction of apoptosis as well as catabolism. On the contrary, overexpression of FBXO21 accelerated cartilage degeneration. Furthermore, FBXO21 performed their effects on cartilage degeneration *via* suppression of autophagy by phosphorylating ERK in chondrocytes (47). Strikingly, JUNB directly targeted the promoter of FBXO21 and upregulated the expression of FBXO21, thus contributing to cartilage degeneration in chondrocytes and SW1353 cells. In a word, JUNB/FBXO21/ERK pathway governs cartilage degeneration *via* blockade of autophagy in osteoarthritis, indicating that downregulation of FBXO21 might be a novel approach for osteoarthritis treatment (47). Jia et al. reported that circRNA-MSR inhibited miR-761 expression and consequently upregulated the expression of FBXO21. FBXO21 and circRNA-MSR expression levels were increased in osteoarthritis, while miR-761 expression levels were decreased in osteoarthritis (92). Depletion of circRNA-MSR facilitated the autophagy of LPS-stimulated cells. Upregulation of FBXO21 abolished the induced autophagy by miR-761 overexpression in chondrocytes. Hence, FBXO21 plays a potential role in osteoarthritis development (92).

### Other E3 ligases

The E3 ubiquitin ligase HECTD1 (HECT domain E3 ubiquitin protein ligase 1) has been proposed to control autophagy and osteoarthritis pathogenesis *via* degradation of Rubicon (48). The expression of HECTD1 was remarkably reduced in patients with osteoarthritis compared with healthy cartilage tissues. Surgery- and aging-triggered osteoarthritis pathogenesis was largely enhanced in cartilage with conditional depletion of HECTD1. Consistently, upregulation of HECTD1 alleviated pathogenesis of osteoarthritis in mouse joints. HECTD1 can interact with Rubicon at K534 and cause ubiquitination and proteasomal degradation of Rubicon, contributing to attenuation of stress-mediated chondrocyte death and osteoarthritis progression (48). Casitas B-lineage lymphoma b (Cbl-b) E3 ubiquitin ligase has been demonstrated to regulate cancer immunotherapy (93). One group identified that Cbl-b targeted Tropomyosin-related kinase A (TrkA) for ubiquitination and degradation (94). It has been known that TrkA is a one of nerve growth factors, which participates in the osteoarthritis pain (95). Knee osteoarthritis that was induced by DMM (destabilization of the medial meniscus) exhibited the dissociation of TrkA and Cbl-b E3 ligase in dorsal root ganglion (DRG) neurons, leading to impaired Cbl-b-mediated degradation of TrkA, thereby contributing to TrkA-dependent pain sensitization (49). In line with this result, overexpression of Cbl-b reduced the levels of TrkA protein and attenuated heat hyperalgesia and mechanical allodynia in DRG neurons of mice with osteoarthritis (49).

Ubiquitin-fold modifier 1-specific ligase 1 (UFL1) E3 ligase had a lower expression level in articular tissues of osteoarthritis. IL-1β treatment alleviated the expression of UFL1 in chondrocytes. Increased expression of UFL1 promoted viability of IL-1β-mediated chondrocytes, which was accompanied with inhibition of NO and PGE2 production and suppression of iNOS and COX-2 expression levels (50). IL-6 and TNF-α levels were elevated after IL-1β induction, which was abrogated by UFL1. Moreover, UFL1 reduced the production of ADAMTS-4, ADAMTS-5, MMP-3 and MMP-13 and inactivated the NF-κB signaling pathway in IL-1β-mediated chondrocytes (50). 3-Hydroxy-3-methylglutaryl reductase degradation (HRD1) E3 ubiquitin ligase exhibits a key role in endoplasmic reticulum (ER)-associated degradation (ERAD) *via* regulating unfolded protein response (UPR) to maintain cellular proteostasis after stress stimulation (96). HRD1 deficient cells had the upregulation of OS9 expression, which was required for degradation of ERAD substrates. HRD1 levels were inversely associated with OS9 expression in clinical synovium tissues of osteoarthritis and rheumatoid arthritis patients (51).

## The role of deubiquitination in osteoarthritis

USPs act as deubiquitinating enzymes, which function to remove Ub from Ub conjugates and control the ubiquitination and degradation of proteins. It has been known that USP play a vital role in bone and bone-related diseases (34). For example, one study identified that a total of 463 ubiquitinated peptides were associated with injured articular cartilage tissues after mechanical injury. These ubiquitinated proteins participated in endoplasmic reticulum (ER)-related protein degradation, such as USP5, YOD1 deubiquitinase, BRCA1/BRCA2-containing complex subunit 3 (BRCC3) E3 ligase, Ataxin 3 (ATXN3) deubiquitinating enzyme. The ER stress regulators were also altered in injured articular cartilage, including ubiquilin 1, RAD23 and VCP/p97 (97). ER stress markers were also elevated after cartilage injury. This work suggested that activation of some DUBs and ER stress regulators involved in regulation of cartilage tissue injury and osteoarthritis (97). Herein, we describe the molecular insight of USPs into osteoarthritis processes (**Table 2**).

**Table 2:** USP regulate osteoarthritis progression.

Name	Targets	Functions	Ref
USP3	SIRT3	Reduces chondrocyte senescence	(98)
USP5	TRAF6	Promotes proinflammatory cytokine production	(99)
USP7	NOX4, SOX9	Controls osteoarthritis progression	(100, 101)
USP13	PTEN/AKT	Inhibits osteoclastogenesis, synovial hyperplasia, cartilage damage, inflammation	(102)
USP14	IκBα	Influences chondrocyte dedifferentiation	(103)
USP15	ERK2	Suppresses osteoarthritis progression	(104)
USP49	Axin	Regulates chondrocyte apoptosis	(105)

### USP3

Ubiquitin-specific protease 3 (USP3) has been reported to inhibit type I interferon signaling *via* deubiquitination of RIG-I-like receptors and antiviral immunity (106). USP3 was found to be downregulated in osteoarthrosis. USP3 upregulation attenuated IL-1β-mediated apoptosis of chondrocytes and inactivation of NF-κB pathway (107). TRAF6 is an adaptor protein for NF-κB pathway and involves in immune response and inflammation. IL-1β elevated the ubiquitination of TRAF6, which can be abrogated by overexpression of USP3 in chondrocytes (107). Increased USP3 expression recued IL-1β-induced cell senescence in rat primary chondrocytes (98). USP3 maintained the protein levels of SIRT3 *via* inhibition of SIRT3 ubiquitination. Consistently, suppression of SIRT3 attenuated the function of USP3 upregulation in cell senescence in rat chondrocytes (98). Similarly, upregulation of SIRT3 abolished USP3-depletion-mediated ROS production and cell senescence. Mechanistically, upregulation of SIRT3 reduced chondrocyte senescence in part *via* FOXO3 deacetylation (98).

### USP5

Evidence has suggested that USP5 is critical involved in inflammation development and processes. For example, USP5 regulated neuropathic and inflammatory pain *via* promotion of CaV3.2 channel activity (108). Impairment of USP5 and Cav3.2 interaction attenuated inflammatory and neuropathic pain (109). A cell-permeant peptide targeting the cUBP domain of USP5 abolished neuropathic pain and inflammatory (110). IL-1β was an essential mediator to regulate the interaction between Cav3.2 and USP5 in the pain process (111). Impairing the binding between USP5 and Cav3.2 protected mechanical hypersensitivity in female mice with peripheral inflammation (112). USP5 was reported to be related with proinflammatory function in RA-FLS (rheumatoid arthritis-fibroblast-like synoviocytes (99). It has been known that RA is a common chronic autoimmune inflammatory disease. USP5 expression was increased in RA-FLS, while the expression of USP5 was decreased in OA-FLS. USP5 expression was increased after IL-1β stimulation in a time-dependent way (99). Overexpression of USP5 aggravated activation of NF-κB pathway and promoted proinflammatory cytokine production. In addition, depletion of USP5 reduced the cytokines release and inactivated NF-κB activation. USP5 can bind with TRAF6 and stabilize TRAF6 *via* deubiquitination. USP5 overexpression controls inflammatory processes *via* maintenance of TRAF6 stability in RA-FLS (99).

### USP7

One study revealed that hydrogen peroxide (H_2_O_2_) induced the expression of USP7 and increased ROS levels and reduced proliferation in rat chondrocytes. Depletion of USP7 abolished H_2_O_2_-mediated pyroptosis and ROS induction and inactivated NLRP3 inflammasome activation in rat chondrocytes (100). Consistently, upregulation of USP7 enhanced pyroptosis, IL-1β and IL-18 levels, MMP-1, MMP13, and NLRP3 inflammasome activation *via* upregulation of ROS levels in rat chondrocytes. Moreover, USP7 interacted with NOX4 and promoted its ubiquitination in rat chondrocytes (100). In addition, USP7 and NOX4 were highly expressed in osteoarthritis patients. Suppression of NOX4 abrogated the function of USP7-mediated pyroptosis, ROS induction and NLRP3 activation in rat chondrocytes. P22077, one USP7 inhibitor, repressed osteoarthritis process in mice with monosodium iodoacetate (MIA) injection. Hence, USP7 regulated NOX4/ROS/NLPR3 pathway to contribute to osteoarthritis progression (100). Ubiquitination of LKB1 increased activation of AMPK pathway and repressed NLRP3 inflammasome response and blocked chondrocyre pyroptosis in osteoarthritis (113).

USP7 expression was decreased in the knee joint cartilage of mice with osteoarthritis. Silencing of USP7 by siRNAs or its inhibitors recued cell proliferation and accelerated apoptosis in chondrocyte. Depletion of USP7 reduced inflammatory response during inflammation process (114). Inhibition of USP7 by its inhibitors promoted cartilage destruction in osteoarthritis mice *via* activation of the BiP-eIF2α-ATF4-CHOP pathway in ERS and promotion of NF-κB/p65 pathway. Several compounds, including QNZ and 4-PBA, and CHOP siRNAs decreased USP7 expression and contributed to inhibition of chondrocyte proliferation and induction of apoptosis and reduction of inflammatory response after TNF-α stimulation (114). One group found that ADMA promoted SOX9 destabilization, which was mediated by DDAH1 in osteoarthritis. DDAH1 expression was decreased, while DDAH1 was increased in osteoarthritis patients (101). Because DDAH1 was an ADMA hydrolase, mice with global or chondrocyte-conditional knockdown of DDAH1 displayed a rapid development of osteoarthritis. ADMA promoted osteoarthritis progression through induction of degeneration and senescence and disruption of ECM deposition in chondrocytes. ADMA interacted with SOX9 and USP7, protecting the SOX9 deubiquitination by USP7 and resulting in promotion of SOX9 degradation. Hence, upregulation of DDAH1 to inhibit ADMA levels and regulate USP7-mediated SOX9 deubiquitination could be a useful strategy for the treatment of osteoarthritis (101).

### USP13

USP13 has been known to participate in the pathogenesis of infection, cancers, inflammation and neurodegenerative diseases (115). For example, USP13 inhibited lung inflammation *via* stabilization of the anti-inflammatory receptor IL-1R8/single immunoglobin interleukin-1-related receptor (Sigirr) (116). Inhibition of miR-19a-3p reduced sepsis-mediated lung injury through upregulation of USP13 expression (117). USP13 impaired the symptoms of lipopolysaccharides-mediated sepsis *via* deubiquitination of IRAK4 (118). USP13 inhibited sepsis-induced inflammation and cardiomyocyte oxidative stress *via* induction of nuclear factor erythroid 2-related factor 2 (Nrf2) (119). One work showed that USP13 regulated PTEN to ameliorate osteoarthritis *via* reducing oxidative stress, regulating apoptosis and inflammation (102). Specifically, USP13 expression was increased in synovial specimens from RA patients. Interestingly, USP13 expression was decreased in human-FLSs after stimulation by LPS, TNF-α and IL-1β. Upregulation of USP13 repressed inflammatory response in H-FLSs upon TNF-α or IL-1β challenge due to the improvement of PTEN and reduction of AKT phosphorylation as well as inactivation of NF-κB pathway (102). USP13 performed the protective functions due to the upregulation of Nrf-2 and downregulation of caspase-3. Mechanistically, USP13 bound with PTEN and regulated AKT activation. Moreover, upregulation of USP13 suppressed the expression of osteoclast-related genes and repressed osteoclastogenesis. In collagen-mediated arthritis (CIA) mice, USP13 attenuated synovial hyperplasia, cartilage damage and inflammation and bone loss (102).

### USP14

USP14 has been known to regulate protein degradation and associate with carcinogenesis, neurodegenerative diseases and viral infections (120, 121). Li et al. reported that USP14 exacerbated activation of NF-κB signaling pathway and accelerated IL-1β-induced chondrocyte dedifferentiation *via* modulation of IκBα degradation (103). USP14 was highly elevated in osteoarthritis articular cartilage and IL-1β-induced chondrocytes. ACHP, an inhibitor of IKK-β, inactivated NF-κB pathway and abrogated USP14 upregulation. In turn, USP14 enhanced IκBα deubiquitination and promoted NF-κB activation. Suppression of NF-κB abolished USP14-mediated dedifferentiation effect of IL-1β on chondrocytes (103). Hence, USP14 might be a useful target for osteoarthritis intervention.

### USP15

USP15 has been uncovered to participate in several cellular processes and tumorigenesis as well as other noncancer diseases (122). USP15 plays the multifaceted roles in various diseases *via* regulating diverse signaling pathways by deubiquitination of target proteins (123). In addition, USP15 has been implied to involve in immune and inflammatory processes by regulation of several pathways, including TLR signaling, NF-κB, RIG-1 pathway, TGF-β, and p53 pathways (124). USP15 was found to stimulate TGF-β/SMAD2 signaling. Moreover, USP15 was required for ERK2 to affect TGF-β/SMAD2 signaling and to control the cartilage phenotype. Mechanistically, USP15 interacted with ERK2 and form a complex to govern the ubiquitination of ERK2. USP15 elevated the levels of phosphor-ERK1/2, but not ERK2 stability, to activate the TGF-β/SMAD2 signaling pathway. Overall, USP15 suppressed osteoarthritis progression *via* targeting ERK and TGF-β/SMAD2 signaling (104).

### USP49

Ubiquitin-specific protease 49 (USP49) has been reported to govern the tumorigenesis. For instance, Fbxo45 promoted cell proliferation and invasion *via* induction of USP49 for ubiquitination and degradation (125). USP49 targets Yes-associated protein 1 (YAP1) for maintaining the stability of YAP1 in gastric cancer, leading to induction of cell proliferation, metastasis, chemoresistance (126). USP49 controlled the oncogenic ability of glucocorticoid receptor beta in glioblastoma cells (127). USP49 was reported to regulate rat chondrocyte apoptosis *via* governing Axin deubiquitination (105). In osteoarthritis patients, the expression of USP49 was lower compared with normal healthy persons. IL-1β stimulation on primary rat chondrocytes caused the downregulation of USP49 expression. Consistently, overexpression of USP49 reduced chondrocyte apoptotic death that was induced by IL-1β stimulation *via* enhancement of Axin deubiquitination, leading to upregulation of Axin protein levels. The increased Axin protein led to β-catenin degradation and suppression of Wnt/β-catenin pathway (105).

## Targeting ubiquitination to regulate osteoarthritis

Numerous compounds have been discovered to target ubiquitination and regulate osteoarthritis (**Table 3**). Digoxin has been approved by FDA to treat several medical conditions, such as heart failure, atrial fibrillation, supraventricular tachycardia, and cardiomyopathy (133, 134). One study showed the effects of digoxin on the inflammatory microenvironment and chondrogenesis in osteoarthritis (128). Digoxin was found to alleviate osteoarthritis synovitis *via* suppression of the M1-like polarization of synovial macrophages in patients with osteoarthritis. The similar results were found in collagenase-mediated osteoarthritis mice (128). By analysis of exosomes produced by macrophages and M1-like macrophages after digoxin treatments, this study found that digoxin governed inflammatory microenvironment of osteoarthritis and enhanced chondrogenesis *via* inhibition of the synovial macrophage M1-like polarization and exosomal miR-146b-5p, USP3 and SOX5 pathways in osteoarthritis (128).

**Table 3:** Compounds target ubiquitination in osteoarthritis.

Name	Targets	Functions	Ref
Digoxin	USP3, SOX5, miR-146b-5p	Regulates inflammatory microenvironment of osteoarthritis, enhances chondrogenesis, inhibits synovial macrophage M1-like polarization.	(128)
6-gingerol	USP49, Wnt/β-catenin	Protects against osteoarthritis	(105)
Resveratrol	IL-1β, caspase-3, PARP, IκBα	Inhibits apoptosis, reduces inflammatory signaling in chondrocytes	(129)
N-Ac-Leu-norleucinal	IκBα	Decreases chondrocyte apoptosis	(129)
5-azacytidine	TGF-β, BMP, Smurf2, Smads	Promotes chondrocyte maturation	(130)
Spermidine	RIP1, CYLD, TNF-α, NF-κB	Performs anti-inflammatory effects on osteoarthritis	(131)
Alpinetin	TNF-α, IκB, MMP13, ADAMTS-5, COL2A1, ERK	Attenuates cartilage matric degradation	(132)

It has been known that [6]-gingerol protected against osteoarthritis *via* regulation of a number of factors, such as Nrf2 and GSTA4-4 (135, 136). One group showed that [6]-gingerol, an anti-osteoarthritis compound, increased the protein levels of USP49 and inhibited the activation of the Wnt/β-catenin signaling pathway in primary rat chondrocytes (105). Resveratrol was reported to inhibit IL-1β-mediated apoptosis *via* regulation of caspase-3 activation and PARP cleavage, and reduce inflammatory signaling *via* inhibition of the degradation of IκBα in articular chondrocytes, suggesting that resveratrol might be a natural compound for treating osteoarthritis (129). ALLN (N-Ac-Leu-norleucinal), the proteasome inhibitor, was also found to repress the degradation of IκBα in chondrocytes and decreased chondrocyte apoptosis (129). 5-azacytidine promoted maturation *via* modulation of TGF-β (transforming growth factor-beta) and BMP (bone morphogenetic protein) pathways in articular chondrocytes (130). 5-azacytidine increased the expression of Smurf2 and decreased the protein levels of Smad2 and Smad3, but elevated the expression of Smad1 and Smad5 as well as inactivation of TGF-β in articular chondrocytes (130).

Spermidine, a natural inducer of autophagy, performs antiaging, anti-inflammation and antitumor activities (137). Spermidine exhibits anti-inflammatory effects on osteoarthritis *via* attenuation of synovitis, cartilage degeneration and osteophyte formation (131). Spermidine facilitated RIP1 deubiquitination by CYLD to block TNF-α-mediated NF-κB signaling pathway in osteoarthritis (131). Alpinetin, a natural flavonoid compound, displayed antitumor, anti-inflammation and antibacterial ability (138). Alpinetin attenuated the TNF-α-mediated upregulation of MMP-13 and ADAMTS-5 and downregulation of COL2A1 expression. Alpinetin also inhibited p65 nuclear translocation and inactivated IκB phosphorylation and regulated ERK phosphorylation. In DMM-induced rats, alpinetin abolished cartilage matrix degradation (132).

## Conclusions and discussions

**Figure 2:**
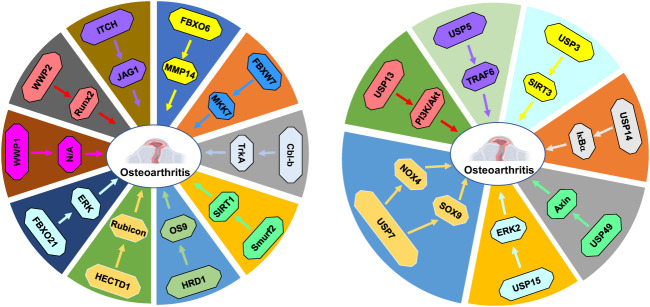
E3 ubiquitin ligases and DUBs in osteoarthritis progression. E3 ubiquitin ligases and DUBs regulate protein ubiquitination and degradation to participate in osteoarthritis development and progression.

In conclusion, ubiquitination and deubiquitination play a potential role in osteoarthritis progression *via* regulating protein degradation (**Figure 2**). Although E3 ubiquitin ligases and DUBs have demonstrated to modulate osteoarthritis processes, several issues should be mentioned. In some studies, the researchers reported the role of ubiquitination in osteoarthritis pathogenesis. However, these studies did not point out the E3 ubiquitin ligases or deubiquitinases. For example, RUNX2 stability was regulated by lncRNAs LINC02035 and LOC100130207, leading to hypertrophic changes in human chondrocytes (139). Depletion of these two lncRNAs facilitated ubiquitin-involved proteasomal degradation of RUNX2 and attenuated hypertrophic differentiation of chondrocytes. Overexpression of LINC02035 and LOC100130207 promoted the stability of RUNX2 protein and induced hypertrophic changes. Therefore, LINC02035 and LOC100130207 might be potential targets for blocking osteoarthritis development *via* delaying chondrocyte hypertrophy (139).

Besides lncRNAs, circRNAs are also involved in osteoarthritis progression (140, 141). Circ_DHRS3 sponged miR-183-5p and elevated the expression of GREM1, resulting in influencing IL-1β-induced chondrocyte proliferation, apoptosis and ECM degradation (142). Circ-9119 intercepted the miR-26a/PTEN axis and prevented chondrocyte apoptosis after IL-1β treatment (143). Circ_0134111 impaired the interaction between miR-224-5p and CCL1 and enhanced osteoarthritis progression (144). CircSERPINE2 protected IL-1β−induced apoptosis and ECM degradation *via* modulation of miR-495/TGFBR2 pathway in chondrocytes (145). CircFNDC3B regulated miR-525-5p and HO-1 pathways and subsequently governed osteoarthritis and oxidative stress (146).

In addition, m6A (N^6^-methyladenosine) modification has been known to regulate osteoarthritis (147–149). METTL3, an enzyme promoted the m6A formation on the mRNA, modulated inflammatory response and apoptosis and increased osteoarthritis development (150). METTL3 affected ECM degradation and governed the inflammatory response, leading to osteoarthritis progression (151). METTL3 reduced the chondrocyte autophagy and apoptosis *via* regulation of Bcl-2 stability by YTHDF1-involved m6A modification (152). METTL3 induced ATG7 m6A modification and enhanced cellular senescence and osteoarthritis progression *via* targeting autophagy and GATA4 pathways (153). METTL3 stimulated LINC00680 expression and facilitated osteoarthritis *via* SIRT1 m6A modification (154). AC008 was increased after m6A modification, leading to accelerating osteoarthritis progression *via* modulation of miR-328-3p and AQP1/ANKH pathways (155). One study implicated that m6A (N^6^-methyladenosine)-modified circRNA RERE regulated osteoarthritis *via* control of β-catenin ubiquitination and degradation (156).

One group found that knockdown of FBXO32 did not alter cartilage destruction in mouse osteoarthritis, although FBXO32 was elevated in osteoarthritis cartilage, suggesting that FBXO32 was not necessary for cartilage destruction in mouse osteoarthritis (157). Besides osteoarthritis, E3 ubiquitin ligases have been revealed to regulate rheumatoid arthritis pathogenesis. For example, E3 ubiquitin ligase TRIM32 (tripartite motif protein 32) overexpression accelerated the production of pro-inflammatory cytokines in FLS of patients with rheumatoid arthritis (158). The expression of TRIM32 was higher in FLS of rheumatoid arthritis patients than that in FLS of osteoarthritis patients. TRIM32 activated NF-κB pathway and bound to TRAF2 to induce the K63-linked polyubiquitination of TRAF2 in rheumatoid arthritis FLS (158). CUL1 affected rheumatoid arthritis *via* alteration of lymphocyte signal transduction (159). E3 ubiquitin ligase STUB1/CHIP regulated the ubiquitination of aryl hydrocarbon receptor and led to the imbalance between Treg and Th17 in rheumatoid arthritis (160).

Like ubiquitination, other PTMs, such as acetylation, methylation, phosphorylation and SUMOylation, have also participated in osteoarthritis progression. Aging increased superoxide dismutase 2 acetylation, which was dependent on Sirtuin 3 in cartilage, leading to osteoarthritis progression (161). GDF11 (growth differentiation factor 11) suppressed the abnormal adipogenesis of chondrocytes and reduced TMJ condylar cartilage degeneration *via* modulation of PPARγ SUMOylation in cartilage of osteoarthritis (162). Desumoylation of aggrecan and collagen II by SENP2 triggered osteoarthritis after IL-1β treatment (163). SIRT1 maintained chondrocyte ECM upon activation of SOX9 *via* deacetylation of FOXO4 (164). Acetylation attenuated SOX9 nuclear accumulation and transactivation of ACAN gene in chondrocytes (161). Protein arginine methyltransferase PRMT1 regulated AKT/FOXO1 pathway and enhanced ECM degradation and apoptosis of chondrocytes in joint osteoarthritis (165). The activity of PRMT1 was elevated and controlled osteoarthritis *via* induction of DHX9 arginine methylation and activation of NF-κB signaling pathway (166). Suppression of PRMT5 reduced MAPK and NF-κB signaling pathway and blocked cartilage degradation in osteoarthritis (167).

There are cross-talks between several PTMs. For instance, SIRT1 restoration regulated PTEN-involved EGFR ubiquitination and accelerated chondrocyte autophagy in osteoarthritis (168). SIRT1 depletion diminished the acetylation of PTEN and then elevated the expression of PTEN. Inactivation of PTEN alleviated EGFR ubiquitination and aggravated EGFR expression *via* destabilization of the EGFR-Cbl complex, contributing to suppression of extracellular matrix degradation in cartilage and promotion of autophagy in chondrocyte (168). In addition, it is necessary to describe that ubiquitin conjugating enzymes involved in osteoarthritis pathogenesis (169, 170). For example, miR-101a-3p increased apoptosis of chondrocytes *via* targeting FZD4 and ubiquitin-conjugating enzyme 2D1 (UBE2D1), leading to involvement of pathogenesis of temporomandibular joint osteoarthritis (169). Ubiquitin conjugating enzyme E2 M (UBE2M) induced apoptosis of chondrocyte *via* upregulation of Wnt/β-catenin signaling in osteoarthritis (170). Without a doubt, it is pivotal to further explore the mechanisms of ubiquitination and deubiquitination in involvement of osteoarthritis development.

## Author contributions

CZ searched the literature and drafted the original manuscript. JC, YW, XW, and YL made the figures and tables and edited the manuscript. LS and WL corrected and edited the manuscript. PW conceived and supervised this study and edited the manuscript. All authors contributed to the article and approved the submitted version.
